# Virtual Visits With Own Family Physician vs Outside Family Physician and Emergency Department Use

**DOI:** 10.1001/jamanetworkopen.2023.49452

**Published:** 2023-12-27

**Authors:** Lauren Lapointe-Shaw, Christine Salahub, Peter C. Austin, Li Bai, R. Sacha Bhatia, Cherryl Bird, Richard H. Glazier, Lindsay Hedden, Noah M. Ivers, Danielle Martin, Jennifer Shuldiner, Sheryl Spithoff, Mina Tadrous, Tara Kiran

**Affiliations:** 1 University Health Network, Toronto, Ontario, Canada; 2 ICES, Toronto, Ontario, Canada; 3 Institute of Health Policy, Management and Evaluation, University of Toronto, Toronto, Ontario, Canada; 4 Department of Medicine, University of Toronto, Toronto, Ontario, Canada; 5 Women’s College Institute for Health System Solutions and Virtual Care, Women’s College Hospital, Toronto, Ontario, Canada; 6 Division of General Internal Medicine and Geriatrics, University Health Network and Sinai Health System, Toronto, Ontario, Canada; 7 Sunnybrook Research Institute, Toronto, Ontario, Canada; 8 Department of Cardiology, University Health Network, Toronto, Ontario, Canada; 9 Patient Partner, Toronto, Ontario, Canada; 10 Department of Family and Community Medicine, University of Toronto, Toronto, Ontario, Canada; 11 MAP Centre for Urban Health Solutions, St Michael’s Hospital, Toronto, Ontario, Canada; 12 Faculty of Health Sciences, Simon Fraser University, Burnaby, British Columbia, Canada; 13 Department of Family Medicine, Women’s College Hospital, Toronto, Ontario, Canada; 14 Women’s College Research Institute, Women’s College Hospital, Toronto, Ontario, Canada; 15 Leslie Dan Faculty of Pharmacy, University of Toronto, Toronto, Ontario, Canada; 16 Department of Family and Community Medicine, St Michael’s Hospital, Unity Health Toronto, Toronto, Ontario, Canada

## Abstract

**Importance:**

Virtual visits became more common after the COVID-19 pandemic, but it is unclear in what context they are best used.

**Objective:**

To investigate whether there was a difference in subsequent emergency department use between patients who had a virtual visit with their own family physician vs those who had virtual visits with an outside physician.

**Design, Setting, and Participants:**

This propensity score–matched cohort study was conducted among all Ontario residents attached to a family physician as of April 1, 2021, who had a virtual family physician visit in the subsequent year (to March 31, 2022).

**Exposure:**

The type of virtual family physician visit, with own or outside physician, was determined. In a secondary analysis, own physician visits were compared with visits with a physician working in direct-to-consumer telemedicine.

**Main Outcome and Measure:**

The primary outcome was an emergency department visit within 7 days after the virtual visit.

**Results:**

Among 5 229 240 Ontario residents with a family physician and virtual visit, 4 173 869 patients (79.8%) had a virtual encounter with their own physician (mean [SD] age, 49.3 [21.5] years; 2 420 712 females [58.0%]) and 1 055 371 patients (20.2%) had an encounter with an outside physician (mean [SD] age, 41.8 [20.9] years; 605 614 females [57.4%]). In the matched cohort of 1 885 966 patients, those who saw an outside physician were 66% more likely to visit an emergency department within 7 days than those who had a virtual visit with their own physician (30 748 of 942 983 patients [3.3%] vs 18 519 of 942 983 patients [2.0%]; risk difference, 1.3% [95% CI, 1.2%-1.3%]; relative risk, 1.66 [95% CI, 1.63-1.69]). The increase in the risk of emergency department visits was greater when comparing 30 216 patients with definite direct-to-consumer telemedicine visits with 30 216 patients with own physician visits (risk difference, 4.1% [95% CI, 3.8%-4.5%]; relative risk, 2.99 [95% CI, 2.74-3.27]).

**Conclusions and Relevance:**

In this study, patients whose virtual visit was with an outside physician were more likely to visit an emergency department in the next 7 days than those whose virtual visit was with their own family physician. These findings suggest that primary care virtual visits may be best used within an existing clinical relationship.

## Key Points

**Question:**

Does health care use differ after a virtual visit with a patient’s own family physician compared with a visit with an outside physician?

**Findings:**

In this cohort study among 5 229 240 Ontario residents with a family physician and virtual visit, patients who had a virtual visit with an outside physician were 66% more likely to visit the emergency department within 7 days compared with those with a virtual visit with their own physician.

**Meaning:**

This finding suggests that virtual care with an outside physician is associated with more emergency department visits.

## Teaser

This cross-sectional study investigates differences in rates of emergency department use among patients who had virtual visits with their own vs outside family physicians.

## Introduction

The COVID-19 pandemic has fueled the growth of virtual care throughout the health care system.^1,2,3,4,5,6,7^ This has included direct-to-consumer telemedicine (also known as virtual-only walk-in clinics), which typically offers on-demand virtual health care without an option for an in-person exam and is disconnected from a patient’s existing source of primary care.^8^ Although some argue that direct-to-consumer telemedicine met a gap in patient care needs,^9^ it also amplified tensions between continuity and access to timely or convenient care.^8^ Furthermore, the ease of access afforded by on-demand virtual care could drive demand for in-the-moment health care that otherwise would not exist.^10^ Some studies have found that direct-to-consumer telemedicine was associated with decreases in health care use by replacing in-person care,^11,12^ while others suggest that it was associated with increased total use and costs.^13,14,15,16,17^

Continuity is a cornerstone of good primary care, and numerous studies have demonstrated that high relational continuity is associated with better health outcomes and lower costs.^18,19,20,21,22^ However, little research has been done to understand differences in subsequent health care use when virtual primary care is provided in the context of an ongoing relationship vs outside of that relationship, as is the case for direct-to-consumer telemedicine.

We used population-based administrative data from Ontario, Canada, to compare subsequent health care use after a virtual visit with a patient’s own family physician vs an outside family physician. We hypothesized that virtual primary care delivered outside an existing relationship would be associated with increased emergency department use.

## Methods

The use of data in this cohort study is authorized under section 45 of Ontario’s Personal Health Information Protection Act and did not require review by a research ethics board or informed consent. We followed the Strengthening the Reporting of Observational Studies in Epidemiology (STROBE) reporting guideline for cohort studies.

### Study Design and Setting

We conducted a population-based, retrospective, propensity score–matched cohort study of residents in Ontario, Canada, who had a virtual visit with a family physician from April 1, 2021, to March 31, 2022. Ontario is Canada’s most populous province, with more than 14.5 million residents. Provincial health insurance is provided to all permanent residents without premiums or copayments and covers emergency department visits, hospitalizations, and all medically necessary physician care. Most primary care is provided by family physicians, and nearly 80% of the population is enrolled to a family physician working in a patient enrollment model.^23^ Enrolling practices are expected to meet all primary care needs, including after-hours care.^24^ In capitation-based models (the model for 65% of enrolling physicians), there is negation (reduction in an access bonus) when patients of enrolling physicians visit family physicians outside the enrolling group.^25^

After the onset of the COVID-19 pandemic in 2020, the Ministry of Health introduced several new, temporary physician billing codes for synchronous virtual visits conducted by video or phone, with a fee equivalent to that of in-person visits. Since then, most publicly funded virtual visits have been conducted by phone.^26,27^ Asynchronous visits, provided by email or text message, were not covered by provincial insurance.

### Data Sources

Population-based health administrative data sets (eTable 1 in Supplement 1) were linked using unique encoded identifiers and analyzed at ICES in Ontario, Canada. ICES is an independent, nonprofit research institute whose legal status under Ontario health information privacy law allows the institute to collect and analyze health care and demographic data without consent for health system evaluation and improvement.

### Study Population

We included all Ontarians who were enrolled to a family physician practicing in a patient enrollment model as of April 1, 2021. We excluded individuals with missing Rurality Index of Ontario scores (a measure of urban or rural residence) or neighborhood income quintile information (<1% missing).^28^

### Exposure

The exposure (a binary variable) was determined at the first virtual family physician visit (ie, index) that was with the patient’s own enrolling physician or with another physician outside the enrolling group from April 1, 2021, to March 31, 2022. We excluded virtual visits with family physicians who worked in a focused practice or with an emergency medicine subspecialization.^29^

We did not include virtual visits with another physician within the same group given that we sought to contrast highest-continuity virtual visits (own physician) with lowest-continuity virtual visits (outside of group). Virtual family physician visits outside the enrolling group likely represented encounters with direct-to-consumer telemedicine clinics (also known as virtual-only walk-in clinics) or combined virtual and in-person walk-in clinics.

### Outcomes

The primary outcome was the occurrence of an emergency department visit within 7 days of the index virtual encounter. Secondary outcomes were a low-acuity emergency department visit (defined as a Canadian Triage and Acuity Scale [CTAS] score of 4-5) within 7 days, an emergency department visit within 30 days, a time to emergency department visit within 30 days, and an in-person or virtual visit with any family physician, the same physician, or own enrolling physician within 7 days. Post hoc outcomes were emergency department visits on day 1 and day 2, a high-acuity emergency department visit within 7 days (CTAS score, 1-2), and a tracer outcome of an emergency department visit for a high-acuity (CTAS score, 1-2) motor vehicle accident on days 3 to 30.^30^ This tracer was chosen given that the acuity level and separation from the index virtual visit by 3 days made it unlikely that such an event would be a cause or consequence of the type of virtual visit.

### Other Variables

We included the following patient characteristics (see operational definitions in eTable 2 in Supplement 1): age, sex, urban or rural residence,^31^ neighborhood income quintile, and whether the patient was a recent insurance registrant (within the past 10 years), a proxy for recent immigration.^23^ We also reported the type of primary care enrollment model, visits to the enrolling physician in the previous 2 years, and the burden of comorbidities using the Johns Hopkins ACG System version 10 resource utilization bands (RUBs), which are based on diagnoses and health care use over the previous 2 years.^32,33^ Related to the index visit, we measured the diagnosis (top 20 categorized), the calendar quarter, whether the visit was on a weekend, if an after-hours code was claimed, and whether the visit was by phone or video or was missing this information (a required code to identify phone or video was introduced in October 2021).

### Statistical Analysis

#### Main Analysis

We first described the cohort using mean (SD), median (IQR), and counts and frequencies. We compared characteristics between groups in unmatched and matched cohorts using standardized mean differences (SMDs), with a difference of 0.1 (10%) or greater considered meaningful.^34^

We derived propensity scores using a logistic regression model that included age, sex, neighborhood income quintile, recent immigrant status, count of visits with the patient’s own physician in the previous 2 years, RUB, enrollment model, diagnosis, modality, and whether the visit was on a weekend or after hours on a weekday. Age and count of visits were modeled using restricted cubic splines with 5 knots at the 5th, 27.5th, 50th, 72.5th, and 95th percentile values.^35^ We then matched exposure groups 1:1 within a caliper distance of 0.2 × the SD of the logit of the propensity score,^36^ while also hard matching on Rurality Index of Ontario category (3 categories: 0-9, large urban; 10-40, small urban; and ≥40, rural) and age group (0-17, 18-64, and ≥65 years).

We reported relative risks (RRs) and risk differences (RDs) for all binary outcomes with 95% CIs, accounting for the paired nature of the matched sample.^37,38^ For the time-to-event outcome, we reported the hazard ratio obtained using a Cox proportional hazards model with robust variance estimate.^39^ Analyses were executed in SAS statistical software version 9.4 (SAS Institute) using a 2-tailed *P* < .05 statistical significance threshold.

#### Subgroup and Sensitivity Analyses

We report primary outcome results for each hard-matched subgroup: patients residing in large urban, small urban, or rural areas and those aged 0 to 17 years, 18 to 64 years, and 65 years or older. We compared the association between type of virtual visit and our tracer outcome to the *e* value,^40^ which is the minimum strength of association that an unmeasured confounder would need to have with the exposure and the outcome to fully explain the observed exposure-outcome association.^41,42^

To separately assess outcomes associated with low-continuity virtual visits without the possibility of a physical examination, we compared virtual visits to a patient’s own physician with visits to a known direct-to-consumer telemedicine clinic. We identified these visits using group billing numbers, a method we updated^13^ for this study (eTable 3 in Supplement 1). Notably, this is a convenience sample based on clinics that use group billing numbers; it is unknown what proportion of all direct-to-consumer telemedicine is covered by this definition.

## Results

Of 5 229 240 individuals in our cohort (eFigure 1 in Supplement 1), 4 173 869 patients (79.8%) had their index virtual encounter with their own physician (mean [SD] age, 49.3 [21.5] years; 2 420 712 females [58.0%]) and 1 055 371 patients (20.2%) had their visit with an outside physician (mean [SD] age, 41.8[20.9] years; 605 614 females [57.4%]). Before matching, patients who had an outside-physician virtual encounter were younger, more often lived in a large urban area (859 772 patients [81.5%] vs 3 207 285 patients [76.8%]; SMD, 0.11), made fewer visits to their own physician in the prior 2 years (mean [SD] 3.2 [4.8] visits vs 7.0 [7.0] visits; SMD, 0.64), were less often enrolled to a non–team capitation model (331 770 patients [31.4%] vs 1 457 804 patients [34.9%]; SMD, 0.13), and more often had visits on weekends (107 945 patients [10.2%] vs 156 859 patients [3.8%]; SMD, 0.26) and using video (14 251 patients [1.4%] vs 9792 patients [0.2%]; SMD 0.13) compared with patients who had an own physician encounter (eTable 4 in Supplement 1).

After matching, there were no differences in measured characteristics of 942 983 matched pairs (total 1 885 966 patients) exceeding 10% (SMD of 0.1) (Table 1; eTable 5 and eFigure 2 in Supplement 1). For example, the mean (SD) age was 42.3 (21.1) years in the outside physician group and 42.5 (20.8) years in the own physician group (SMD, 0.01). Patients who had a virtual visit with an outside physician saw that physician a median (IQR) of 0 (0-2) times in the 2 years prior to the index visit compared with 2 (0-5) visits with their own physician.

**Table 1.** Patient Characteristics in Matched Cohort

Characteristic^a^	Patients with virtual encounter, No. (%) (N = 1 885 966)		SMD
	Physician outside enrolling group (n = 942 983)	Own enrolling physician (n = 942 983)	
Age, y			
Mean (SD)	42.3 (21.1)	42.5 (20.8)	0.01
Median (IQR)	41 (26-58)	41 (27-58)	0.01
Sex			
Female	539 878 (57.3)	540 301 (57.3)	<0.01
Male	403 105 (42.7)	402 682 (42.7)	<0.01
Area of residence			
Large urban	756 053 (80.2)	756 053 (80.2)	<0.01
Small urban	143 298 (15.2)	143 298 (15.2)	<0.01
Rural	43 632 (4.6)	43 632 (4.6)	<0.01
Neighborhood income quintile			
1 (Lowest)	168 887 (17.9)	165 539 (17.6)	0.01
2	181 198 (19.2)	180 345 (19.1)	<0.01
3	197 497 (20.9)	196 497 (20.8)	<0.01
4	198 298 (21.0)	199 231 (21.1)	<0.01
5 (Highest)	197 103 (20.9)	201 371 (21.4)	0.01
Recent registrant in Ontario	89 691 (9.5)	86 533 (9.2)	0.01
Primary care enrolment model type			
Team capitation	232 919 (24.7)	241 343 (25.6)	0.02
Nonteam-based capitation	311 859 (33.1)	317 326 (33.7)	0.03
Enhanced fee for service	391 653 (41.5)	378 914 (40.2)	0.01
Other group	6552 (0.7)	5400 (0.6)	0.02
Visits with own enrolling physician in previous 2 y			
Mean (SD)	3.6 (4.9)	3.7 (4.8)	0.01
Median (IQR)	2 (0-5)	2 (0-5)	0.04
Resource Utilization Band			
High	133 276 (14.1)	128 140 (13.6)	0.02
Moderate	492 726 (52.3)	498 016 (52.8)	0.01
Low	316 981 (33.6)	316 827 (33.6)	<0.01
Quarter of index virtual encounter			
Q1 (April to June)	449 009 (47.6)	449 330 (47.6)	<0.01
Q2 (July to September)	225 766 (23.9)	229 628 (24.4)	0.01
Q3 (October to December)	147 735 (15.7)	144 723 (15.3)	0.01
Q4 (January to March)	120 473 (12.8)	119 302 (12.7)	<0.01
Type of virtual encounter at index			
Phone	249 335 (26.4)	250 336 (26.5)	<0.01
Video	9344 (1.0)	7382 (0.8)	0.02
Missing	684 304 (72.6)	685 265 (72.7)	<0.01
Index virtual encounter day			
On a weekend	84 649 (9.0)	76 058 (8.1)	0.03
Claimed with after-hours code on a weekday	35 445 (3.8)	34 101 (3.6)	0.01

Abbreviation: SMD, standardized mean difference.

^a^ Not listed: top 20 diagnoses (eTable 5 in Supplement 1).

### Emergency Department Visits

Patients who had a virtual encounter with an outside physician were 66% more likely to visit an emergency department within 7 days (30 748 patients [3.3%] vs 18 519 patients [2.0%]; RD, 1.3% [95% CI, 1.2%-1.3%]; RR, 1.66 [95% CI, 1.63-1.69]) (Table 2). This corresponds to 1 additional emergency department visit for every 77 outside virtual visits. The increase in risk associated with virtual visits with outside vs own physicians was greater for the outcome of low-acuity (7759 patients [0.8%] vs 4084 patients [0.4%]; RR, 1.90 [95% CI, 1.83-1.97]) than high-acuity (7042 patients [0.7%] vs 4836 patients [0.5%]; RR, 1.46 [95% CI, 1.40-1.51]) emergency department visits within 7 days.

**Table 2.** Patient Outcomes in Matched Cohort

Outcome	Patients with virtual encounter, No. (%) (N = 1 885 966)		RD, % (95% CI)	RR (95% CI)
	Physician outside enrolling group (n = 942 983)	Own enrolling physician (n = 942 983)		
ED visit within 7 d				
Any	30 748 (3.3)	18 519 (2.0)	1.3 (1.2-1.3)	1.66 (1.63-1.69)
High acuity	7042 (0.7)	4836 (0.5)	0.2 (0.2-0.3)	1.46 (1.40-1.51)
Low acuity	7759 (0.8)	4084 (0.4)	0.4 (0.4-0.4)	1.90 (1.83-1.97)
ED visit				
Day 1	12 661 (1.3)	6372 (0.7)	0.7 (0.6-0.7)	1.99 (1.93-2.05)
Day 2	6566 (0.7)	3539 (0.4)	0.3 (0.3-0.3)	1.86 (1.78-1.93)
Within 30 d	57 674 (6.1)	41 342 (4.4)	1.7 (1.7-1.8)	1.40 (1.38-1.41)
Mean (SD)	8.9 (9.1)	10.4 (9.2)	NA	HR = 1.41 (1.39-1.43)
ED visit for high-acuity motor vehicle accident day 3-30^a^	129 (<0.1)	97 (<0.1)	<0.1	1.33 (1.02-1.73)
In-person visit within 7 d				
With any family physician	57 208 (6.1)	45 828 (4.9)	1.2 (1.1-1.3)	1.25 (1.23-1.26)
With same physician	29 043 (3.1)	39 102 (4.1)	1.1 (1.0-1.1)	0.74 (0.73-0.75)
With own enrolling physician	9915 (1.1)	39 102 (4.1)	3.1 (3.1-3.1)	0.25 (0.25-0.26)
With physician in own group	11 532 (1.2)	38 994 (4.1)	2.9 (2.9-3.0)	0.30 (0.29-0.30)
Virtual visit within 7 d				
With any family physician	83 681 (8.9)	44 470 (4.7)	4.2 (4.1-4.2)	1.88 (1.86-1.90)
With same physician	40 100 (4.3)	39 251 (4.2)	0.1 (0.0-0.2)	1.02 (1.01-1.04)
With own enrolling physician	19 658 (2.1)	39 251 (4.2)	2.1 (2.0-2.1)	0.50 (0.49-0.51)
With physician in own group	20 924 (2.2)	38 882 (4.1)	1.9 (1.9-2.0)	0.54 (0.53-0.55)

Abbreviations: ED, emergency department; HR, hazard ratio; NA, not applicable; RD, risk difference; RR, relative risk.

^a^ Canadian Triage and Acuity Scale score, 1 to 2.

The increased risk of an emergency department visit was front-loaded, with an RR of 1.99 (95% CI, 1.93-2.05) on day 1 and 1.86 (95% CI, 1.78-1.93) on day 2 and early separation of survival curves (eFigure 3 in Supplement 1). However, there was still an association at 30 days (57 674 patients [6.1%] vs 41 342 patients [4.4%]; RR, 1.40 [95% CI, 1.38-1.41]).

### Family Physician Visits

Patients who had an outside-physician virtual encounter were more likely than those with an own physician visit to have an in-person family physician visit within 7 days (57 208 patients [6.1%] vs 45 828 patients [4.9%]; RR, 1.25 [95% CI, 1.23-1.26]) but were less likely to have such a visit with their own physician (9915 patients [1.1%] vs 39 102 patients [4.2%]; RR, 0.25 [95% CI, 0.25-0.26]). Similarly, they were nearly twice as likely to have a virtual visit within 7 days (83 681 patients [8.9%] vs 44 470 patients [4.7%]; RR, 1.88 [95% CI, 1.86-1.90]), and this was also less likely to be with their own physician (19 658 patients [2.1%] vs 39 251 patients [4.2%]; RR, 0.50 [95% CI, 0.49-0.51]).

### Subgroup Analyses

The increase in risk of a 7-day emergency department visit associated with having an outside-physician virtual visit was greater for younger age groups. Children and adolescents (ages <18 years) were at highest risk (RR, 1.96 [95% CI, 1.86-2.05]), followed by adults (ages 18-64 years; RR, 1.69 [95% CI, 1.65-1.73]) and older adults (ages ≥65 years; RR, 1.40 [95% CI, 1.34-1.45]) (eTable 6 in Supplement 1). There were no differences between patients by residence in large urban, small urban, or rural areas.

### Sensitivity Analyses

The type of virtual visit was also associated with our tracer outcome of emergency department visits for high-acuity motor vehicle accidents, with a higher risk for those who visited an outside physician (129 people [0.01%]) than those who visited their own physician (97 people [0.01%]; RD, 0%; RR, 1.33 [95% CI, 1.02-1.73]). This association was weaker than the confounder strength of association (*e* value^41^ lower confidence limit = 2.32) that would be needed to explain away the findings for our primary outcome.

We used an alternative exposure definition (eTable 7 in Supplement 1) that compared a visit with a patient’s own physician with a visit with a known direct-to-consumer telemedicine clinic. In this analysis, 30 216 patients with a direct-to-consumer telemedicine visit were nearly 3 times more likely to visit the emergency department within 7 days than 30 216 patients with own physician visits (1878 patients [6.2%] vs 628 patients [2.1%]; RD, 4.1% [95% CI, 3.8%-4.5%]; RR, 2.99 [95% CI, 2.74-3.27]). Similar to findings in the main cohort, the increased risk was front-loaded in the first 2 days (eTable 8 and eFigure 4 in Supplement 1). Direct-to-consumer telemedicine users were more likely to have a repeat virtual visit within 7 days than those with a virtual visit to their own physician (3191 patients [10.6%] vs 1105 patients [3.7%]; RR, 2.89 [95% CI, 2.70-3.09]), although this was not explained by visits with the same physician, their own physician, or another physician in their group.

## Discussion

In this population-based cohort study, we compared outcomes of patients who received a virtual visit with their own family physician with outcomes of those who received a virtual visit with a family physician outside their physician group. We found that the latter patients had more visits to the emergency department in the ensuing 7 days, although the absolute difference was small, at 1.3%, corresponding to 1 additional emergency department visit for every 77 visits outside the group. We found that patients who had a visit with a subset of direct-to-consumer telemedicine clinics had approximately 3 times the risk of 7-day emergency department visits and repeat virtual visits than those who had a virtual visit with their own physician.

The increased use of the emergency department associated with low-continuity virtual visits was front-loaded in the first few days, suggesting that virtual visits may serve a triaging function, allowing for the identification of patients who would benefit from an in-person assessment. Direct-to-consumer telemedicine physicians may direct patients to an in-person follow-up visit, either immediately or if symptoms persist; alternatively, physicians may direct patients to the emergency department for a condition perceived to be urgent. Finally, some patients may choose the emergency department even if not suggested by a physician if they perceive this to be their only option for a timely in-person exam.

Patients having virtual visits outside the enrolling group were also more likely to have repeat virtual or in-person family physician visits, although these were less commonly with their enrolling physician, suggesting that these visits may have been repeat visits to walk-in clinics or virtual walk-in clinics. These patients may have faced challenges or barriers to accessing their usual physician or group, thus leading them to seek care elsewhere, including through a virtual walk-in clinic or an emergency department. Delays to an appointment, even for individuals already attached to a family physician, have been well described in the US and Canada.^43,44^ However, visits with multiple outside clinicians may be associated with increased costs and fragmented care.^45^ Care fragmentation increases the risk for errors and can introduce inconsistent and sometimes conflicting messages, eroding trust in physicians and the health care system as a whole.^18,46,47,48^ Achieving timely access while preserving care continuity remains an important health policy challenge.

Our study is novel given that we directly contrasted outcomes after high- and low-continuity forms of virtual care. Meanwhile, previous research compared virtual visits with in-person visits. Within-system virtual care (integrated with in-person, office-based care) from US-based Intermountain Healthcare and Kaiser Permanente has compared more favorably with in-person care^49,50^ than has direct-to-consumer telemedicine, which has been associated with increased downstream health care use and costs.^14,15,17,51^ In 2 Ontario studies,^52,53^ patients whose regular family physicians provided more virtual care did not have higher rates of emergency department use. Furthermore, within-system virtual care has demonstrated the potential for associated increases in equity of care^54^ via improved access for individuals who struggle to afford higher visit-related costs.^55,56,57^

Some health systems in the US and Europe have adopted integrated virtual care models.^49,54,58^ However, other insurers, such as US Medicare, the UK National Health Service (NHS), and the Canadian provinces of Nova Scotia and British Columbia, have contracted out publicly insured health care services to corporate virtual clinics.^59,60,61,62^ In a time series–based evaluation of privately insured residents of Minnesota,^12^ plan members with coverage for direct-to-consumer telemedicine had lower costs of care for episodes of urinary tract infection but not sinusitis after plan expansion. An evaluation^63^ of the NHS’s GP at Hand, a program of direct-to-consumer telemedicine supported by in-person locations, found that users had higher rates of consultations than the general population, despite being younger and in better health. This raised concerns about inequities given that public funding was diverted to the service. Policymakers continue to refine virtual care policies to optimize the balance of access, quality, and costs.

Our findings provide evidence to support policy changes that prioritize virtual visits within an existing therapeutic relationship. These findings complement those of McGrail et al^64^ from a smaller study of patients who received virtual visits in British Columbia, Canada, in 2013 to 2014. In that study, potential cost savings from virtual visits were greater among patients who saw a known physician. We also build on a previous Ontario-based study^13^ in which we found that patients who had visits with virtual walk-in clinics in 2020 were twice as likely as those with other types of virtual family physician visits to visit the emergency department within 30 days. In addition to better addressing potential sources of confounding, this study extends our findings to 2021, when emergency department visit volumes began to rise to their pre-COVID-19 levels.^52^

### Limitations

Our study has several limitations. First, some outside-physician virtual visits may have averted emergency department visits for patients who were not able to access their own physician in a timely way. There is no way to identify such patients or determine which elements of access (eg, hours of operation, availability of physicians, or challenges making appointments) may have contributed. Second, we were limited to studying publicly funded virtual visits given that information on privately funded visits was not available. Third, the association between the type of virtual visit and trauma-related emergency department visits is unlikely to be explained by health care–seeking behavior alone given that we included only the highest-acuity visits. This association could reflect differences between groups in risk-taking behavior and health literacy.^65^ However, confounding on this basis would not be strong enough to explain the association between the type of virtual visit and 7-day emergency department visits. Patients who perceive their care needs to be urgent or who have a lower tolerance for delays may be more likely to seek immediate care through direct-to-consumer telemedicine and emergency departments, another potential source of confounding. Fourth, Ontario residents do not pay premiums or copayments for any visit type included in this study. Therefore, our findings may not be fully generalizable to settings where outside group care is financially disincentivized.

## Conclusions

This cohort study found that for patients attached to a family physician, a virtual visit with an outside physician instead of the patient’s own physician was associated with more emergency department visits and family physician visits in the following week. Our findings support the use of primary care virtual visits within an existing clinician-patient relationship.

## Notes

Supplement 1. **eTable 1.** ICES Data Sources **eTable 2.** Operational Definitions of All Variables **eTable 3.** Search Strategy for Direct-to-Consumer (Virtual-Only Walk-In Clinic) Group Numbers **eTable 4.** Characteristics of Patients in Unmatched Cohort **eTable 5.** Top 20 Diagnoses of All Patients in Matched Cohort **eTable 6.** Relative Risk of Having an Emergency Department Visit After a Virtual Visit With a Physician Outside Patient Enrolling Group by Age and Rurality Subgroup **eTable 7.** Sensitivity Analysis: Characteristics of Patients in the Matched Cohort **eTable 8.** Patient Outcomes in the Matched Sensitivity Cohort **eFigure 1.** Study Population Flowchart **eFigure 2.** Density of Propensity Scores Before and After Matching **eFigure 3.** Kaplan-Meier Curve of Time to Emergency Department Visit for Patients With Index Virtual Visit With Own Enrolling Family Physician and Physician Outside Enrolling Group **eFigure 4.** Kaplan-Meier Curve of Time to Emergency Department Visit for Patients With Index Virtual Visit With Own Enrolling Physician and Virtual-Only Walk-In Clinic
eReferences.

Supplement 2.
Data Sharing Statement
