# Supplementary material for: Virtual Visits With Own Family Physician vs Outside Family Physician and Emergency Department Use
Source: JAMA Netw Open. 2023 Dec 27;6(12):e2349452. doi: 10.1001/jamanetworkopen.2023.49452 (PMC10753397; doi:10.1001/jamanetworkopen.2023.49452)
Supplement: Supplement 1. — eTable 1. ICES Data Sources eTable 2. Operational Definitions of All Variables eTable 3. Search Strategy for Direct-to-Consumer (Virtual-Only Walk-In Clinic) Group Numbers eTable 4. Characteristics of Patients in Unmatched Cohort eTable 5. Top 20 Diagnoses of All Patients in Matched Cohort eTable 6. Relative Risk of Having an Emergency Department Visit After a Virtual Visit With a Physician Outside Patient Enrolling Group by Age and Rurality Subgroup eTable 7. Sensitivity Analysis: Characteristics of Patients in the Matched Cohort eTable 8. Patient Outcomes in the Matched Sensitivity Cohort eFigure 1. Study Population Flowchart eFigure 2. Density of Propensity Scores Before and After Matching eFigure 3. Kaplan-Meier Curve of Time to Emergency Department Visit for Patients With Index Virtual Visit With Own Enrolling Family Physician and Physician Outside Enrolling Group eFigure 4. Kaplan-Meier Curve of Time to Emergency Department Visit for Patients With Index Virtual Visit With Own Enrolling Physician and Virtual-Only Walk-In Clinic eReferences. [file jamanetwopen-e2349452-s001.pdf]

Supplemental Online Content

Lapointe-Shaw L, Salahub C, Austin PC, et al. Virtual visits with own family physician vs outside family physician and emergency department use. *JAMA Netw Open*. 2023;6(12):e2349452. doi:10.1001/jamanetworkopen.2023.49452

eTable 1. ICES Data Sources

eTable 2. Operational Definitions of All Variables

eTable 3. Search Strategy for Direct-to-Consumer (Virtual-Only Walk-In Clinic) Group Numbers

eTable 4. Characteristics of Patients in Unmatched Cohort

eTable 5. Top 20 Diagnoses of All Patients in Matched Cohort

eTable 6. Relative Risk of Having an Emergency Department Visit After a Virtual Visit With a Physician Outside Patient Enrolling Group by Age and Rurality Subgroup

eTable 7. Sensitivity Analysis: Characteristics of Patients in the Matched Cohort

eTable 8. Patient Outcomes in the Matched Sensitivity Cohort

eFigure 1. Study Population Flowchart

eFigure 2. Density of Propensity Scores Before and After Matching

eFigure 3. Kaplan-Meier Curve of Time to Emergency Department Visit for Patients With Index Virtual Visit With Own Enrolling Family Physician and Physician Outside Enrolling Group

eFigure 4. Kaplan-Meier Curve of Time to Emergency Department Visit for Patients With Index Virtual Visit With Own Enrolling Physician and Virtual-Only Walk-In Clinic

eReferences.

This supplemental material has been provided by the authors to give readers additional information about their work.

eTable 1. ICES Data Sources.

Database name	Description
**Primary Care Population (PCPOP)**	Population-level dataset that includes all people in Ontario who are deemed alive and eligible to receive primary care at a given point in time- this relies on any healthcare encounter in Ontario in the previous 8 years. All indicators are as of the index date, with various lookback periods. The dataset is produced twice annually (April 1st and October 1st of each year) and contains information on demographics, primary care enrolment, and healthcare utilization over the previous 12 month period. The version of PCPOP used in the present study was from April 1st 2021.[1]
**Corporate Provider Database (CPBD)**	Information on providers (physicians, nurses, etc.) and groups (primary care, hospitals, etc.) eligible to receive payment from the Ontario Health Insurance Plan (OHIP), such as physician demographics, training, and practice location.[3] This dataset was used to find group billing numbers identifying select virtual-only walk-in clinic practices.
**Discharge Abstract Database (DAD)**	Information on all admissions (excluding designated mental health beds) to acute care hospitals in Ontario. This includes dates of admission as well as diagnostic and procedural codes. Overall, diagnostic codes were found to be 82% sensitive for primary diagnosis when verified against chart abstraction.[4]
**National Ambulatory Care Reporting System (NACRS)**	Includes information for all emergency department visits since 2000. A re-abstraction study of diagnostic codes found 85% agreement for the main presenting problem.[5]
**Ontario Health Insurance Plan (OHIP)**	Contains information on all billing claims submitted by Ontario physicians (consultations, assessments and procedures). Physicians practicing in non fee-for-service models (capitation-based patient enrolment models) submit shadow billings to OHIP, which appear as billing claims with a payment value of $0.[6]

© 2023 Lapointe-Shaw L et al. *JAMA Netw Open.*

eTable 2. Operational Definitions of All Variables.

Variable	Data Source	Definition
Age	PCPOP	AGE in PCPOP
Sex	PCPOP	SEX in PCPOP:<br>Male<br>Female
Urban/rural residence	PCPOP	Postal code converted to RIO score (RIOG in PCPOP[7]):<br>0-9: Large urban<br>10-39: Small urban<br>40+: Rural
Neighborhood income quintile	PCPOP	Nearest census-based income quintile based on postal code (based on 2016 census, INCQUINT in PCPOP). Categories from 1 (lowest) to 5 (highest).
Recent provincial health insurance registrant	PCPOP	Recent registrant within the past 10 years, used as a proxy for immigration (IMMIG in PCPOP). Missing values are children less than 10 years of age.
Primary care enrolment model type	PCPOP	Categorized from PROGTYPE3 in PCPOP as:<br>Team capitation (Family Health Team)<br>Non-team capitation (Family Health Network or Family Health Organization)<br>Enhanced fee-for-service (Family Health Group or Comprehensive Care Model)<br>Other group
Previous visits to own enrolling physician	PCPOP	Count of visits with the patient's own enrolling physician (PCPOP VIS_OWNP) in the previous 2 years.
Resource Utilization Band	DAD, NACRS, OHIP	Using Resource Utilization Bands (RUBs), per the Johns Hopkins ACG® System Version 7, in 2 years prior to the index date.<br>Categorized as:<br>Low: 0-2<br>Moderate: 3<br>High: 4-5
Quarter of index virtual encounter	PCPOP	The calendar quarter in which the virtual encounter occurred, between April 1st 2021 and March 31st 2022.
Type of virtual encounter at index	PCPOP	Using K billing codes (introduced October 1st 2021), each visit was categorized as:<br>Phone (K301)<br>Video (K300)<br>Missing (all others)
Index virtual encounter was on a weekend	PCPOP	Whether the visit was on a Saturday or Sunday (yes/no).
Index virtual encounter was claimed with an	PCPOP	Whether the index virtual visit occurred on a weekday (Monday to Friday) *and* contained any of the following after-hours codes: A888, A994, A996, A998, E409, E410, Q994, Q996, Q998, Q012, Q016, Q017

© 2023 Lapointe-Shaw L et al. *JAMA Netw Open.*

after-hours code on a weekday		
Emergency department (ED) visit within 7 days	NACRS	Any emergency department visit (i.e., all Canadian Triage and Acuity Scale CTAS scores) in the 7 days following the index virtual visit.
Low-acuity ED visit within 7 days	NACRS	Any emergency department visit with an ADMCAT score of 4 or 5 (CTAS score), in the 7 days following the index virtual visit.
High-acuity ED visit within 7 days	NACRS	Any emergency department visit with an ADMCAT score of 1 or 2 (CTAS score), in the 7 days following the index virtual visit.
Emergency department (ED) visit on day 1/day 2/within 30 days	NACRS	Any emergency department visit on:<br>1. The day of the index virtual visit (day 1).<br>2. The day following the index virtual visit (day 2).<br>3. Within 30 days of the index virtual visit.
Time to emergency department visit (days)	NACRS	Median days to any emergency department visit following the index virtual visit, up to 30 days.
Emergency department visit for high-acuity (CTAS 1-2) motor vehicle accident, days 3-30	NACRS	An emergency department visit for a motor vehicle accident with Canadian Triage and Acuity Scale scores of 1 or 2 within 3 to 30 days following the index virtual visit. All diagnosis codes from V00 to V69 in the NACRS dataset were used to indicate a motor vehicle accident.[5,8]
In-person visit with any/same/own enrolling family physician or own group physician – 7 days	OHIP PCPOP	A visit within 7 days of the index virtual visit with location = O (office) or H (home) for at least one code in that encounter, and:<br>1. Any family physician (SPEC = 00, family physician).<br>2. The same family physician as the index virtual visit (OHIP PHYSNUM = PHYSNUM at index visit).<br>3. The patient's own enrolling physician (OHIP PHYSNUM = PHYSNUM in PCPOP).
Virtual visit with any/same/own enrolling family physician or own group physician– 7 days	OHIP PCPOP	A visit within 7 days of the index virtual visit with location = P (virtual) for at least one code in that encounter, and:<br>1. Any family physician (SPEC = 00, family physician).<br>2. The same family physician as the index virtual visit (OHIP PHYSNUM = PHYSNUM at index visit).<br>3. The patient's own enrolling physician (OHIP PHYSNUM = PHYSNUM in PCPOP).

© 2023 Lapointe-Shaw L et al. *JAMA Netw Open.*

eTable 3. Search Strategy for Direct-to-Consumer Telemedicine (Virtual-Only Walk-in Clinic) Group Numbers.

Search	Approach	Result
**1. Group Name Search to Google Search Strategy**- Search was performed in duplicate, and each clinic website was double-checked by CS and JS to confirm eligibility	Using a keyword search in Excel, we examined all group names present in the Canadian Physician Database (CPDB) that included the term 'virtual' or 'tele' (n = 43). We then used Google to search each clinic name, and included clinics that had a working website, offered services to patients in Ontario, and provided virtual appointments only (i.e., no brick-and-mortar clinic for in-person visits nor the option of home visits). We excluded clinics that did not provide primary care (e.g., specialty clinics such as pain or cannabis clinics).	6 virtual-only walk-in clinics.
**2. Google Search to Group Name Strategy**- Search was performed in duplicate, and each clinic website was double-checked by CS and JS to confirm eligibility	Next, we conducted a Google search using the following Boolean search term: *(Canada OR Ontario) AND ("virtual clinic" OR "telemedicine").* We examined the preview of all available pages and found 5 virtual additional walk-in clinics present in the CPDB that met the inclusion criteria above. Three more virtual clinics were included based on our team members' knowledge of virtual walk-in services available in Ontario. To find any additional clinics, we used the Boolean search term created by Matthewman et al. (2021): *(Canada) AND (virtual healthcare OR virtual health) AND (family medicine OR clinic OR general practitioner OR personalized care).* No other virtual clinics from this search met the inclusion criteria (same as step 1 above).	8 virtual-only walk-in clinics.
**3. Active Billings During the Study Period**	Of the 14 virtual walk-in clinics obtained from steps 1 and 2 above, 6 clinics had no billings during the study time period, and we removed 1 clinic because it had only a total of 4 virtual claims in the study period.	7 virtual walk-in clinics for our analyses.

© 2023 Lapointe-Shaw L et al. *JAMA Netw Open.*

eTable 4. Characteristics of Patients in the Unmatched Cohort.

Variables	Virtual encounter with a physician outside the enrolling group N=1,055,371	Virtual encounter with their own enrolling physician N=4,173,869	Standardized Mean Difference (SMD)
**Age**, Mean (SD)	41.8 (20.9)	49.3 (21.5)	0.35
Median (IQR)	40 (26-57)	52 (34-66)	0.37
**Age**, n (%)			
<18 years	136,614 (12.9)	425,279 (10.2)	0.09
19-29	196,763 (18.6)	413,488 (9.9)	0.25
30-44	265,377 (25.1)	797,221 (19.1)	0.15
45-64	287,728 (27.3)	1,409,715 (33.8)	0.14
65-74	96,037 (9.1)	634,983 (15.2)	0.19
75+	72,852 (6.9)	493,183 (11.8)	0.17
**Sex**, n (%)			
Female	605,614 (57.4)	2,420,712 (58.0)	0.01
Male	449,757 (42.6)	1,753,157 (42.0)	0.01
**Area of residence**, n (%)			
Large urban	859,772 (81.5)	3,207,285 (76.8)	0.11
Small Urban	150,476 (14.3)	748,798 (17.9)	0.10
Rural	45,123 (4.3)	217,786 (5.2)	0.04
**Neighbourhood income quintile**, n (%)			
1 (lowest)	196,228 (18.6)	704,136 (16.9)	0.05
2	205,610 (19.5)	796,855 (19.1)	0.01
3	220,478 (20.9)	865,665 (20.7)	0
4	219,142 (20.8)	896,329 (21.5)	0.02
5 (highest)	213,913 (20.3)	910,884 (21.8)	0.04
**Recent registrant** in Ontario, n (%)	98,391 (9.3)	320,910 (7.7)	0.06
**Primary care enrolment model type**, n (%)			
Team capitation	243,178 (23.0)	1,086,966 (26.0)	0.07
Non-team capitation	331,770 (31.4)	1,457,804 (34.9)	0.13
Enhanced fee-for-service	473,554 (44.9)	1,603,074 (38.4)	0.07
Other group	6,869 (0.7)	26,025 (0.6)	0
**Previous visits to own enrolling physician,**			
Mean (SD)	3.2 (4.8)	7.0 (7.0)	0.64
Median (IQR)	1 (0-5)	5 (2-9)	0.84
**Resource Utilization Band**, n (%)			
High	149,231 (14.1)	629,489 (15.1)	0.03
Moderate	555,977 (52.7)	2,342,408 (56.1)	0.07
Low	350,163 (33.2)	1,201,972 (28.8)	0.10

© 2023 Lapointe-Shaw L et al. *JAMA Netw Open.*

**Quarter of virtual encounter,** n (%)			
Q1 (April-June)	500,495 (47.4)	2,406,463 (57.7)	0.21
Q2 (July-September)	250,751 (23.8)	845,719 (20.3)	0.08
Q3 (October-December)	168,284 (15.9)	505,359 (12.1)	0.11
Q4 (January-March)	135,841 (12.9)	416,328 (10.0)	0.09
**Type of virtual encounter**, n (%)			
Phone	272,087 (25.8)	904,926 (21.7)	0.10
Video	14,251 (1.4)	9,792 (0.2)	0.13
Missing	769,033 (72.9)	3,259,151 (78.1)	0.12
**Weekend**, n (%)	107,945 (10.2)	156,859 (3.8)	0.26
**After-hours on a weekday**, n(%)	35,557 (3.4)	410,837 (9.8)	0.26

© 2023 Lapointe-Shaw L et al. *JAMA Netw Open.*

eTable 5. Top 20 Diagnoses of All Patients in the Matched Cohort.

Diagnosis	Virtual encounter with a physician outside the enrolling group, n (%) N=942,983	Virtual encounter with their own enrolling physician, n (%) N=942,983	Standardized Mean Difference (SMD)
1. Anxiety neurosis, hysteria, neurasthenia, obsessive compulsive neurosis, reactive depression	57,748 (6.1)	57,875 (6.1)	0
2. Other ill-defined conditions	82,069 (8.7)	79,257 (8.4)	0.01
3. Essential, benign hypertension	32,985 (3.5)	29,264 (3.1)	0.02
4. Diabetes mellitus, including complications	26,864 (2.8)	24,047 (2.6)	0.02
5. Leg cramps, leg pain, muscle pain, joint pain, arthralgia, joint swelling, masses	33,391 (3.5)	31,837 (3.4)	0.01
6. Anorexia, nausea and vomiting, heartburn, dysphagia, hiccough, hematemesis, jaundice, ascites, abdominal pain, melena, masses	25,748 (2.7)	23,857 (2.5)	0.01
7. Disorders of lipoid metabolism (e.g., hypercholesterolemia, lipoprotein disorders)	17,653 (1.9)	16,794 (1.8)	0.01
8. Immunization - all types	15,355 (1.6)	14,506 (1.5)	0.01
9. Acute nasopharyngitis, common cold	31,497 (3.3)	27,145 (2.9)	0.03
10. Eczema, atopic dermatitis, neurodermatitis	19,307 (2.0)	17,717 (1.9)	0.01
11. Lumbar strain, lumbago, coccydynia, sciatica	12,482 (1.3)	11,612 (1.2)	0.01
12. Other non-specific abnormal findings	13,989 (1.5)	12,705 (1.3)	0.01
13. Convulsions, ataxia, vertigo, headache, except tension headache and migraine	12,073 (1.3)	11,036 (1.2)	0.01
14. Family planning, contraceptive advice, advice on sterilization or abortion	18,096 (1.9)	17,581 (1.9)	0
15. Chest pain, tachycardia, syncope, shock, edema, masses	10,156 (1.1)	9,508 (1.0)	0.01
16. Coronavirus	17,079 (1.8)	16,100 (1.7)	0.01
17. Allergic rhinitis, hay fever	12,618 (1.3)	11,344 (1.2)	0.01
18. Osteoarthritis	9,359 (1.0)	8,791 (0.9)	0.01
19. Other disorders of urinary tract	14,868 (1.6)	13,062 (1.4)	0.02
20. Hypothyroidism - acquired (i.e., myxedema)	9,922 (1.1)	8,657 (0.9)	0.01
Others	469,724 (49.8)	500,288 (53.1)	0.07

© 2023 Lapointe-Shaw L et al. *JAMA Netw Open.*

eTable 6. Relative Risk of Having an Emergency Department Visit After a Virtual Visit with a Physician Outside the Patient's Enrolling Group by Age and Rurality Subgroups.

Subgroup	Relative Risk, 95% CI
Large Urban	1.67 (1.64-1.70)
Small Urban	1.63 (1.57-1.71)
Rural	1.63 (1.51-1.75)
Age 0-17	1.96 (1.86-2.05)
Age 18-64	1.69 (1.65-1.73)
Age 65+	1.40 (1.34-1.45)
Age 0-17 and Large Urban	2.0 (1.90-2.12)
Age 18-64 and Large Urban	1.68 (1.64-1.73)
Age 65+ and Large Urban	1.41 (1.34-1.47)

© 2023 Lapointe-Shaw L et al. *JAMA Netw Open.*

eTable 7. Sensitivity Analysis: Characteristics of Patients in the Matched Cohort*.

Variables	Virtual encounter with a virtual walk-in clinic N=30,216	Virtual encounter with their own enrolling physician N=30,216	Standardized Mean Difference (SMD)
**Age**, Mean (SD)	36.0 (17.8)	35.5 (17.4)	0.03
Median (IQR)	33 (24-47)	33 (24-46)	0.03
**Age**, n (%)			
<18 years	3,663 (12.1)	4,225 (14.0)	0.06
19-29	8,666 (28.7)	8,181 (27.1)	0.04
30-44	9,380 (31.0)	9,716 (32.2)	0.02
45-64	6,167 (20.4)	5,891 (19.5)	0.02
65-74	1,457 (4.8)	1,487 (4.9)	0.01
75+	883 (2.9)	716 (2.4)	0.03
**Sex**, n (%)			
Female	18,344 (60.7)	18,371 (60.8)	0
Male	11,872 (39.3)	11,845 (39.2)	0
**Area of residence**, n (%)			
Large urban	22,202 (73.5)	22,202 (73.5)	0
Small Urban	6,283 (20.8)	6,283 (20.8)	0
Rural	1,731 (5.7)	1,731 (5.7)	0
**Neighbourhood income quintile**, n (%)			
1 (lowest)	5,553 (18.4)	5,500 (18.2)	0.01
2	6,187 (20.5)	6,147 (20.3)	0
3	6,069 (20.1)	6,214 (20.6)	0.01
4	6,313 (20.9)	6,378 (21.1)	0.01
5 (highest)	6,094 (20.2)	5,977 (19.8)	0.01
**Recent registrant** in Ontario, n (%)	2,039 (6.7)	1,883 (6.2)	0.02
**Primary care enrolment model type**, n (%)			
Team capitation	7,851 (26.0)	7,855 (26.0)	0
Non-team capitation	10,987 (36.4)	10,691 (35.4)	0.02
Enhanced fee-for-service	11,136 (36.9)	11,412 (37.8)	0.02
Other group	242 (0.8)	258 (0.9)	0.01
**Previous visits to own enrolling physician,**			
Mean (SD)	3.0 (4.3)	2.9 (4.2)	0.03
Median (IQR)	2 (0-4)	1 (0-4)	0.01
**Resource Utilization Band**, n (%)			
High	3,444 (11.4)	3,215 (10.6)	0.02
Moderate	14,993 (49.6)	15,190 (50.3)	0.01
Low	11,779 (39.0)	11,811 (39.1)	0
**Quarter of virtual encounter,** n (%)			
Q1 (April-June)	13,434 (44.5)	13,528 (44.8)	0.01

© 2023 Lapointe-Shaw L et al. *JAMA Netw Open.*

Q2 (July-September)	7,466 (24.7)	7,689 (25.4)	0.02
Q3 (October-December)	5,192 (17.2)	5,077 (16.8)	0.01
Q4 (January-March)	4,124 (13.6)	3,922 (13.0)	0.02
**Type of virtual encounter**, n (%)			
Phone	7,079 (23.4)	6,877 (22.8)	0.02
Video	2,237 (7.4)	2,122 (7.0)	0.02
Missing	20,900 (69.2)	21,217 (70.2)	0.02
**Weekend**, n (%)	5,726 (19.0)	5,002 (16.6)	0.06
**After-hours on a weekday**, n (%)	70 (0.2)	85 (0.3)	0.01

* Variables in PS model were: age (in years), sex, neighborhood income quintile, recent immigrant status, count of visits with own physician in previous 2 years, resource utilization band, type of enrolment model, diagnosis (top 20, and other), index visit on a weekend, after-hours on a weekday, and modality (phone, video or missing). Age was fitted with a spline using 5 knots at the 5, 27.5, 50, 72.5 and 95th percentile values, and visits were fitted using 3 knots at values of 2, 4 and 8 visits. Match was 99% (30216/30392 observations in the virtual walk-in group were matched).

eTable 8. Patient Outcomes in the Matched Sensitivity Cohort.

Outcome	Virtual encounter with a virtual-only walk-in clinic, n (%) N=30,216	Virtual encounter with their own enrolling physician, n (%) N=30,216	Risk difference, % (95% CI)	Relative Risk (95% CI)
**Emergency department visit within 7 days**	1,878 (6.2)	628 (2.1)	4.1 (3.8-4.5)	2.99 (2.74-3.27)
**High-acuity emergency department visit within 7 days**	345 (1.1)	158 (0.5)	0.6 (0.5-0.8)	2.18 (1.81-2.63)
**Low-acuity emergency department visit within 7 days**	535 (1.8)	150 (0.5)	1.3 (1.1-1.4)	3.57 (2.98-4.27)
**Emergency department visit on day 1**	890 (2.9)	214 (0.7)	2.2 (2.0-2.5)	4.16 (3.59-4.82)
**Emergency department visit on day 2**	382 (1.3)	131 (0.4)	0.8 (0.7-1.0)	2.92 (2.39-3.55)
**Emergency department visit within 30 days**	2,919 (9.7)	1,328 (4.4)	5.3 (4.9-5.7)	2.20 (2.06-2.34)
**Time to emergency department visit (days)**	-	-	-	HR* = 2.27 (2.13-2.42)
**In-person visit with any family physician- 7 days**	1,390 (4.6)	1,109 (3.7)	0.9 (0.6-1.3)	1.25 (1.16-1.35)
**In-person visit with same physician- 7 days**	22 (0.1)	923 (3.1)	3.0 (2.8-3.2)	0.02 (0.02-0.04)
**In-person visit with own enrolling physician-7 days**	463 (1.5)	923 (3.1)	1.5 (1.3-1.8)	0.50 (0.45-0.56)
**In-person visit with own group physician- 7 days**	552 (1.8)	968 (3.2)	1.4 (1.1-1.6)	0.57 (0.51-0.63)
**Virtual visit with any family physician- 7 days**	3,191 (10.6)	1,105 (3.7)	6.9 (6.5-7.3)	2.89 (2.70-3.09)
**Virtual visit with same physician- 7 days**	560 (1.9)	959 (3.2)	1.3 (1.1-1.6)	0.58 (0.53-0.65)
**Virtual visit with own enrolling physician- 7 days**	1,060 (3.5)	959 (3.2)	0.3 (0.1-0.6)	1.11 (1.01-1.20)
**Virtual visit with own group physician- 7 days**	1,178 (3.9)	987 (3.3)	0.6 (0.3-0.9)	1.19 (1.10-1.30)

HR* = Hazard ratio

© 2023 Lapointe-Shaw L et al. *JAMA Netw Open.*

eFigure 1: Study Population Flowchart.

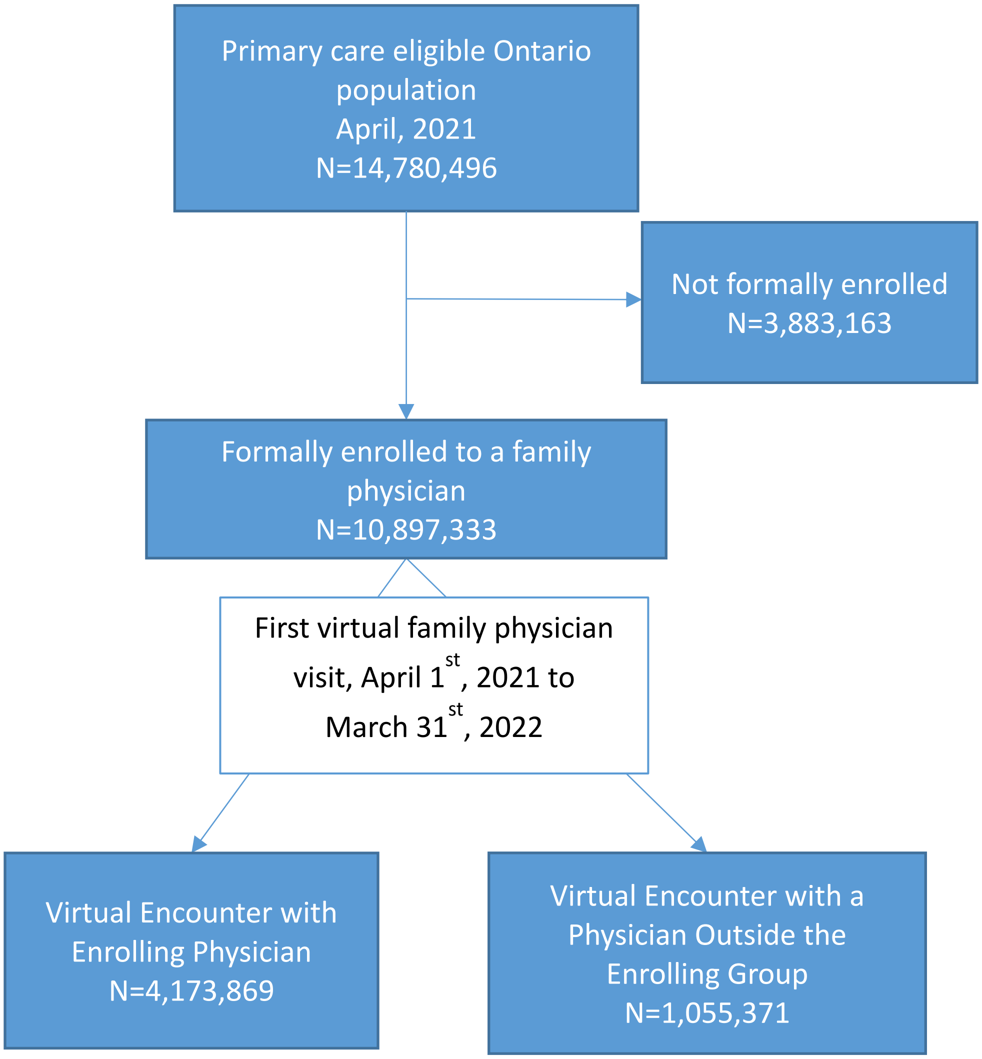

© 2023 Lapointe-Shaw L et al. *JAMA Netw Open.*

eFigure 2. Density of Propensity Scores Before and After Matching.

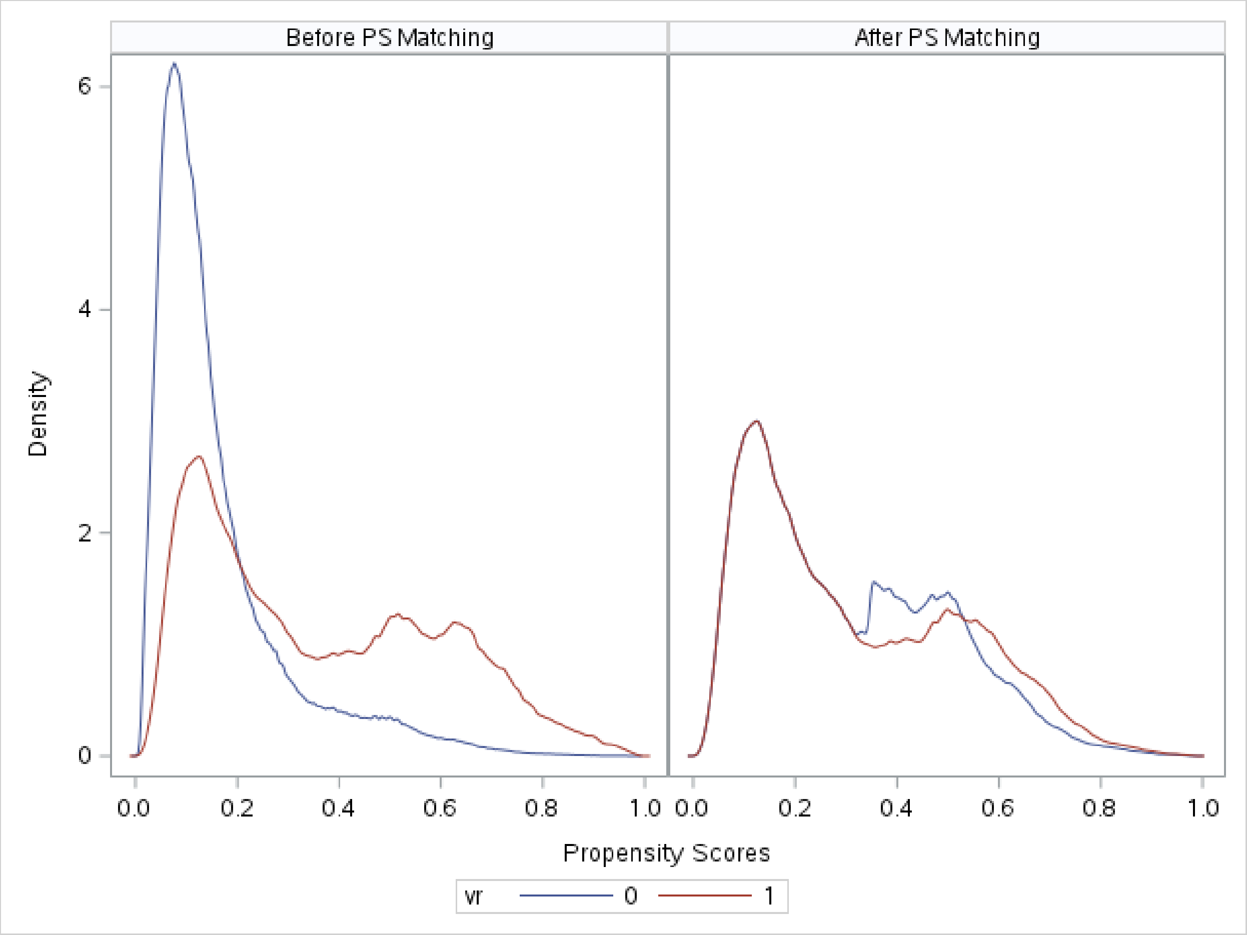

Note. Vr = 0 (blue line), virtual encounter with enrolling physician. Vr = 1 (red line), virtual encounter with a physician outside the enrolling group.

© 2023 Lapointe-Shaw L et al. *JAMA Netw Open.*

eFigure 3: Kaplan-Meier Curve of Time to Emergency Department Visit for Patients with an Index Virtual Visit with their Own Enrolling Family Physician and with a Physician Outside the Enrolling Group.

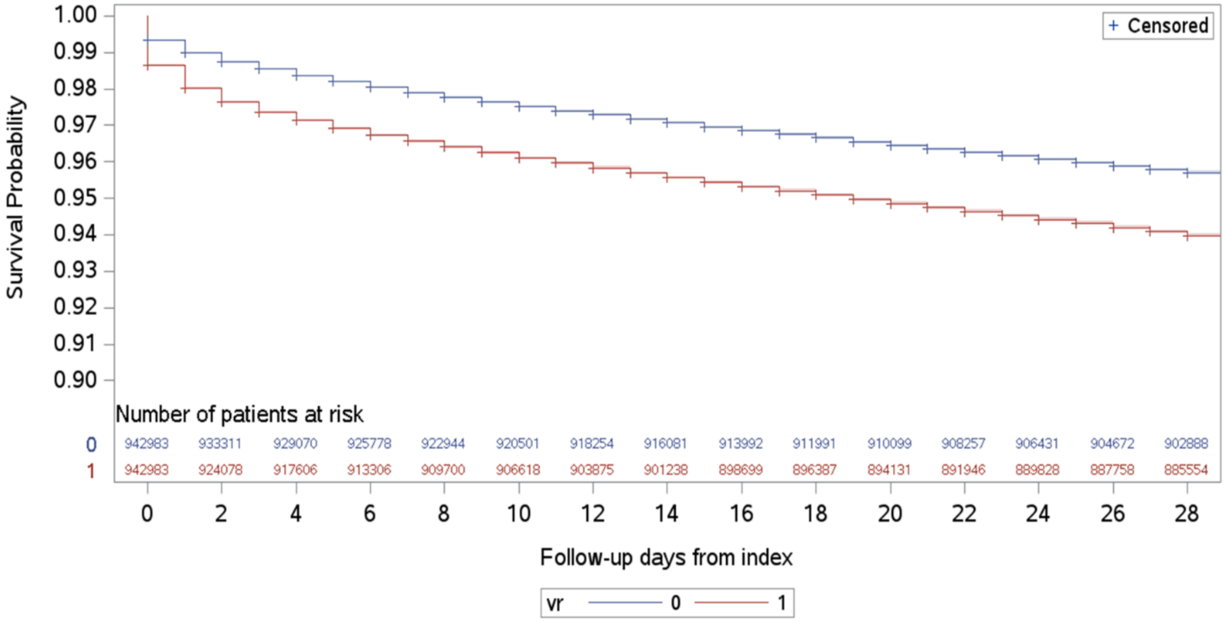

Note: Patients with an index virtual visit with their own enrolling family physician (coded as ‘0’, in blue), and with a physician outside the enrolling group (coded as ‘1’, in red).

© 2023 Lapointe-Shaw L et al. *JAMA Netw Open.*

eFigure 4: Kaplan-Meier Curve of Time to Emergency Department Visit for Patients with an Index Virtual Visit with their Own Enrolling Physician and a Virtual-Only Walk-In Clinic.

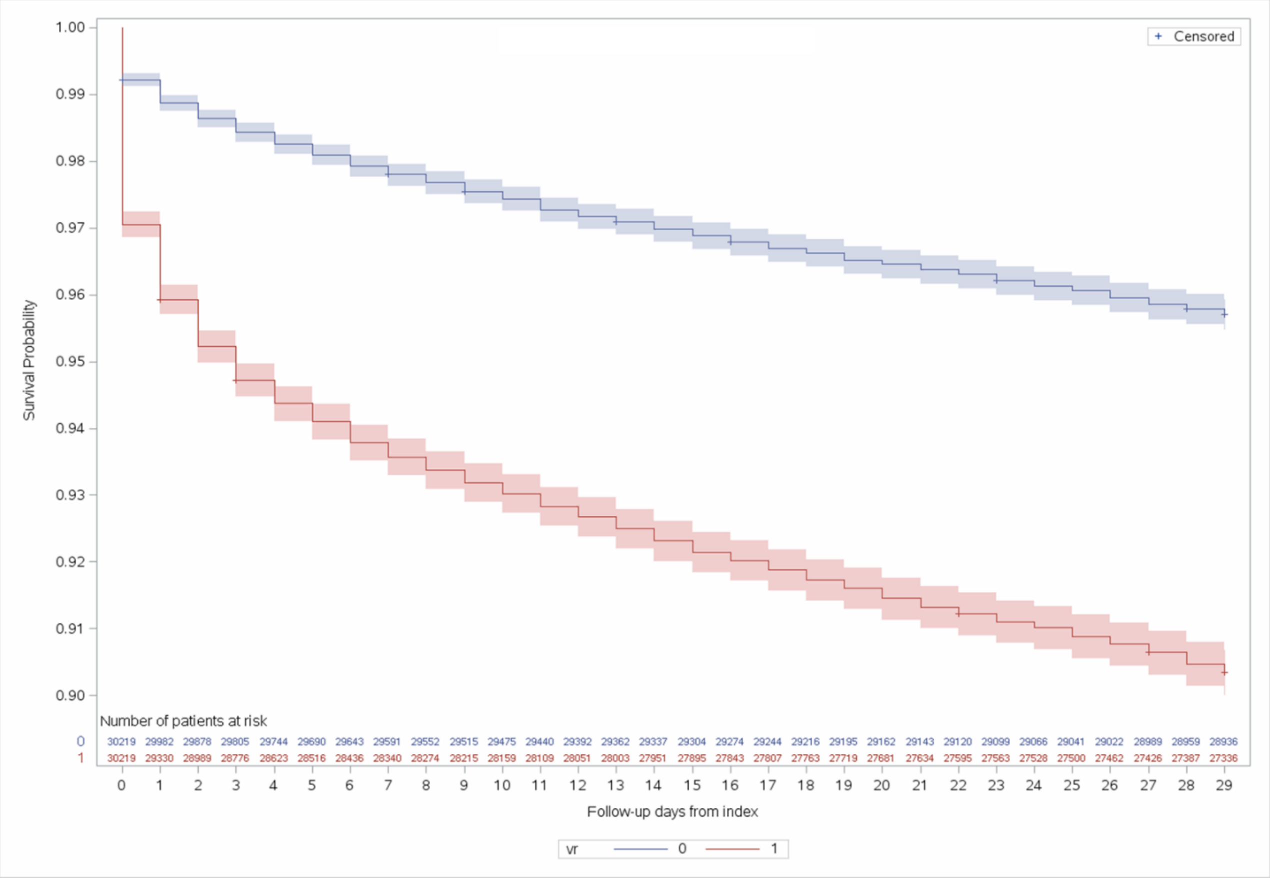

Note: Patients with an index virtual visit with their own enrolling family physician (coded as ‘0’, in blue), and with a virtual-only walk-in clinic (coded as ‘1’, in red).

© 2023 Lapointe-Shaw L et al. *JAMA Netw Open.*

References

1. ICES Data Dictionary for PCPOP. Accessed May 23, 2023. https://datadictionary.ices.on.ca/Applications/DataDictionary/Library.aspx?Library=PCPOP

2. Glazier RH, Kopp A, Schultz SE, Henry TK and DA. All the Right Intentions but Few of the Desired Results: Lessons on Access to Primary Care from Ontario's Patient Enrolment Models. *Healthcare Quarterly*. 2012;15(3).

3. ICES. Corporate Provider Database (CPDB). Accessed December 28, 2022. https://datadictionary.ices.on.ca/Applications/DataDictionary/Library.aspx?Library=CPDB

4. Juurlink D, Preyra C, Croxford R, et al. Canadian Institute for Health Information Discharge Abstract Database: A Validation Study. Published online 2006. Accessed December 28, 2022. https://www.ices.on.ca/Publications/Atlases-and-Reports/2006/Canadian-Institute-for-Health-Information

5. CIHI Data Quality Study of Ontario Emergency Department Visits for Fiscal Year 2004–2005—Executive Summary. Canadian Institute for Health Information. Published online 2007. Accessed December 28, 2022. https://secure.cihi.ca/free_products/vol1_nacrs_executive_summary_nov2_2007.pdf

6. ICES Data Dictionary for OHIP. Accessed May 23, 2023. https://datadictionary.ices.on.ca/Applications/DataDictionary/Library.aspx?Library=OHIP

7. Kralj B. Measuring Rurality - RIO2008_BASIC: Methodology and Results. Accessed October 26, 2021. https://content.oma.org/wp-content/uploads/2008rio-fulltechnicalpaper.pdf

8. Redelmeier DA, Wang J, Thiruchelvam D. COVID Vaccine Hesitancy and Risk of a Traffic Crash. *The American Journal of Medicine*. 2023;136(2):153-162.e5. doi:10.1016/j.amjmed.2022.11.002

© 2023 Lapointe-Shaw L et al. *JAMA Netw Open.*
